# Survey of methods and principles in three-dimensional reconstruction from two-dimensional medical images

**DOI:** 10.1186/s42492-023-00142-7

**Published:** 2023-07-27

**Authors:** Mriganka Sarmah, Arambam Neelima, Heisnam Rohen Singh

**Affiliations:** 1 Department of Computer Science and Engineering, National Institute of Technology, Nagaland, 797103 India; 2 Department of Information Technology, Nagaland University, Nagaland, 797112 India

**Keywords:** Three-dimensional reconstruction, Human organ, Medical images

## Abstract

Three-dimensional (3D) reconstruction of human organs has gained attention in recent years due to advances in the Internet and graphics processing units. In the coming years, most patient care will shift toward this new paradigm. However, development of fast and accurate 3D models from medical images or a set of medical scans remains a daunting task due to the number of pre-processing steps involved, most of which are dependent on human expertise. In this review, a survey of pre-processing steps was conducted, and reconstruction techniques for several organs in medical diagnosis were studied. Various methods and principles related to 3D reconstruction were highlighted. The usefulness of 3D reconstruction of organs in medical diagnosis was also highlighted.

## Introduction

**Fig. 1:**
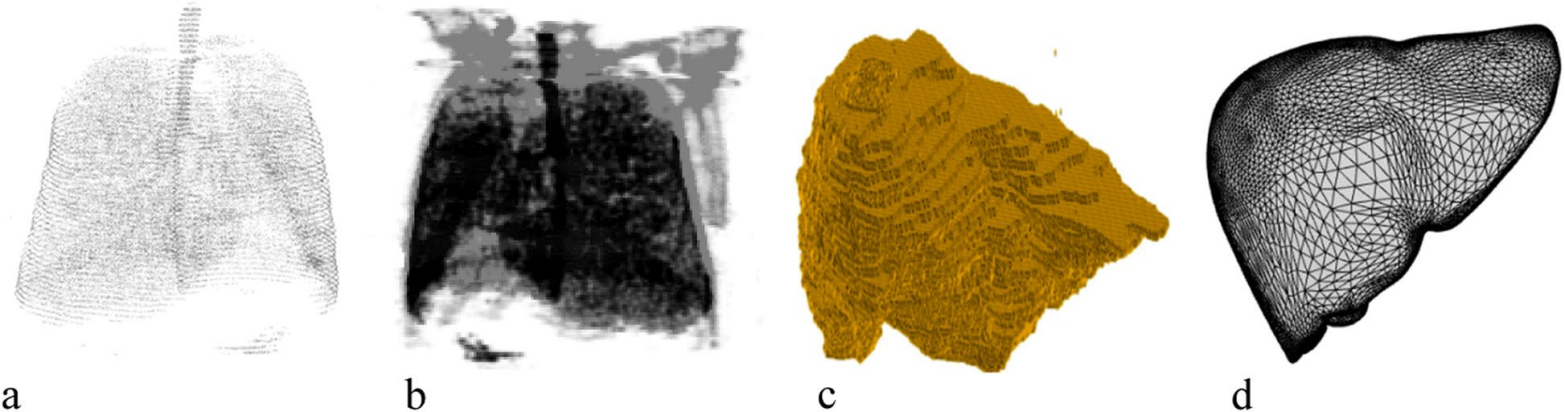
**a** Point cloud from CT of lungs; **b** Point cloud reconstructed from CT scans of rib cage (gray) and lungs (black); **c** Coarse liver reconstructed from 28 CT scans and triangulated using marching cubes algorithm; **d** Smooth liver surface triangulation of key points extracted from 56 CT scans

Three-dimensional (3D) reconstruction is used to create a 3D model of an object or scene from a series of two-dimensional (2D) images (Fig. 1). This process has gained significant attention in recent years owing to its wide range of applications in fields including medicine, entertainment, archaeology, and robotics. 3D reconstruction produces a digital representation of a real-world object. Use of 3D reconstruction in the medical sciences began with the invention of a computed tomography (CT) scanner by Godfrey Hounsfield [1], useful for training, virtual surgery [2], and gaining insight into the behavior of organs in vivo. 3D reconstruction can help in planning and monitoring pre-operative and post-operative medical conditions of patients. Werner et al. [3] constructed a 3D model of the respiratory behavior of the lungs at the inhale and exhale stages. Werner et al. [4] constructed a 3D model to diagnose cervical tumors from ultrasound images using virtual bronchoscopy. 2D medical image formats such as magnetic resonance imaging (MRI), CT, positron emission tomography (PET), x-rays, ultrasound, and microscopy have been used for 3D reconstruction. Of these, ultrasound is noninvasive and harmless. The choice of data acquisition method is a determining factor in the effectiveness of 3D reconstruction algorithms [5]. Hardie et al. [6] used a laser scanning confocal microscope for data acquisition; Zollhöfer et al. [7] used an RGB camera. Østergaard et al. [8] assessed abnormalities in rheumatoid arthritis using radiography, MRI, and ultrasonography in a comparative study.

Radiography is performed only from the posterior-anterior and lateral (LAT) angles; 3D reconstruction based on x-rays [9–14] is challenging because there are only one or two initial images. Reconstruction processes require more images to develop an organ in 3D. Thus, x-ray-based modeling requires a different methodology. Digitally reconstructed radiographs (DRRs) [15] have been used to overcome this limitation. A DRR is generated from multiple CT or MRI scans. The techniques used for 3D reconstruction from 2D x-ray images are discussed in Miscellaneous section.

Methods have been developed to generate a DRR based on statistical shape models (SSMs) [16–19]. These models are constructed from a collection of image data; their shapes and sizes can be heavily altered. SSMs create a mean model from several volumetric reference models. The accuracy of the mean model depends on the number of reference models. With a small number of reference models, the 3D reconstruction model is inaccurate. Methods based on an active contour model (ACM) [20] can be used to reform a poorly constructed SSM [21]. ACM techniques reform the model boundary according to a specified target model using a threshold limit or error metric such as the minimum contour-to-mesh distance. In some cases, an articulated shape model [22] is used, in which the reference shape is a concatenation of shapes that are movable at fixed joints, such as at the point of concatenation (knee, jaw, or hip). To reduce the complexity of handling large-point data, the dimensions of the region of interest (ROI) are reduced using principal component analysis (PCA) [23].

The 3D model can be stored in different formats including WaveFront object, polygon file format, point cloud data, and stereo lithography [9]. Jiang et al. [24] used a point cloud as the input to produce a polygon surface as the output in the structure-from-motion problem [25]. In 3D reconstruction from medical images, generating a point cloud is the first step to reconstruction. All medical imaging-based 3D reconstructions are modeled using a point set [26]; the surface is represented as a mesh of triangles [27] or an octree [28]. In an octree, a point set within a voxel is grouped under a parent node, which is referred to by another parent node within a larger voxel.

Surface reconstruction can be categorized into parametric, implicit, and spatial subdivision methods. In parametric methods [29], the surface is a function of two parameters along the $x$ and $y$ axes. The surface under reconstruction is updated such that its parameters align with the set of target parameters. However, this technique is not suitable for models with no known topological relationships. Another method is implicit reconstruction. The goal of the implicit method [30] is to define a zero set of roots of a function that best fits the target surface, similar to the contour-matching method. The measurements involve a distance function to approximate the closeness of the points. Reconstruction methods based on spatial subdivision techniques [31] were derived from Delaunay triangulation [32] and Voronoi graphs [33]. The point distribution is initially covered by an approximate surface. With further iterations, new points are added by dividing the initial coarse assignment into consecutive finer and smoother surfaces.

## Methods

### Reconstruction based on traditional approach

The reconstruction process comprises three stages: segmentation, registration, and surface reconstruction.

#### Segmentation

Image segmentation and segmentation mask prediction are two common problems in image pre-processing and 3D reconstruction. Thresholding-based methods [34] use a threshold to filter the noise. The output is a binary image with pixels that are either black or white. These methods are suitable for intensity-based region discovery. Region growing (RG) methods [35] start with a seed pixel as a node and join neighboring pixels with similar intensity. Region-merging and splitting [36] intake an entire image and segment it into four sub-images in iterative steps based on a similarity measure between inter-segment metrics until no further segmentation is possible. Clustering-based methods [37] use a distance measure for the intensity values to segment into multiple clusters. A limitation in clustering is that smooth edges and gradual intensity transitions are not easily grouped into non-intersecting clusters. Edge-detection methods [38] use layers of Gaussian filters, changing their sigma values for edge detection. These methods segment the image without understanding the underlying shape information or region semantics.

3D reconstruction methods require a ROI and additional features such as landmark points, curvature values, and angular variation in pairs of edges. Mumford and Shah [39] used a variational model to measure changes within a set of points. It applied a piecewise smooth representation to the image boundary. Getreuer [40] developed an ACM defined over level sets.

These methods are based on minimizing the energy functional $F\left(u,C\right)$ (Eq. 1), where* u* is the set of image points, and *C* is the image contour or boundary. *µ* and *ν* are positive constants; Ω bounds a 2D area, and curve *C* ⊂ Ω.

1 $$F\left(u,C\right)=\int\limits_\Omega^{}\left|u-u_0\right|^2\delta x\delta y+\mu\int\limits_{\Omega\backslash C}^{}{\nabla u}^2\delta x\delta y+\nu\cdot length\left(C\right)$$

Several studies have used level sets [41] (Eq. 2), in which the image is segmented into an inside region *u*^*I*^ and an outside region *u*^*II*^.

2 $$F\left(u^I,u^{II},C\right)=\int\limits_{inside\left(C\right)}^{}\left|u^I-u^O\right|^2\delta x\delta y+\mu\int\limits_{inside\left(C\right)}^{}\left|{\nabla\mathrm u}^{\mathrm I}\right|^2\delta x\delta y+\int\limits_{outside\left(C\right)}^{}\left|u^{II}-u^O\right|^2\delta x\delta y+\mu\int\limits_{outside\left(C\right)}^{}\left|{\nabla\mathrm u}^{\mathrm{II}}\right|^2\delta x\delta y+\nu\cdot length\left(C\right)$$

**Fig. 2:**
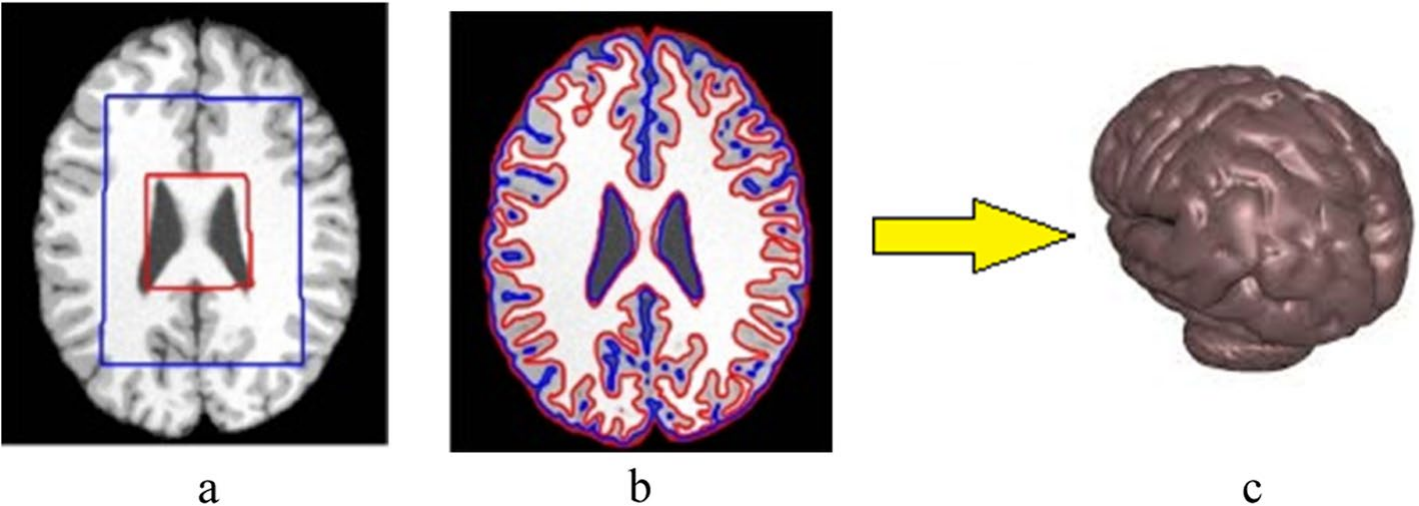
**a** Brain CT; **b** Segmented white matter and gray matter; **c** Reconstructed model

*u*^*I*^ and *u*^*II*^ are functions used to approximate the image *u*^*O*^ inside and outside the curve, respectively. The images obtained for 3D medical reconstruction were mostly 2D CT slices. The images were stacked on top of one another [42]. Figure 2 shows a CT slice segmented for the contour boundary using the snake method after providing an initial seed curve. Figure 2 shows the segmentation process using active contours that change shape based on the gradient.

**Fig. 3:**
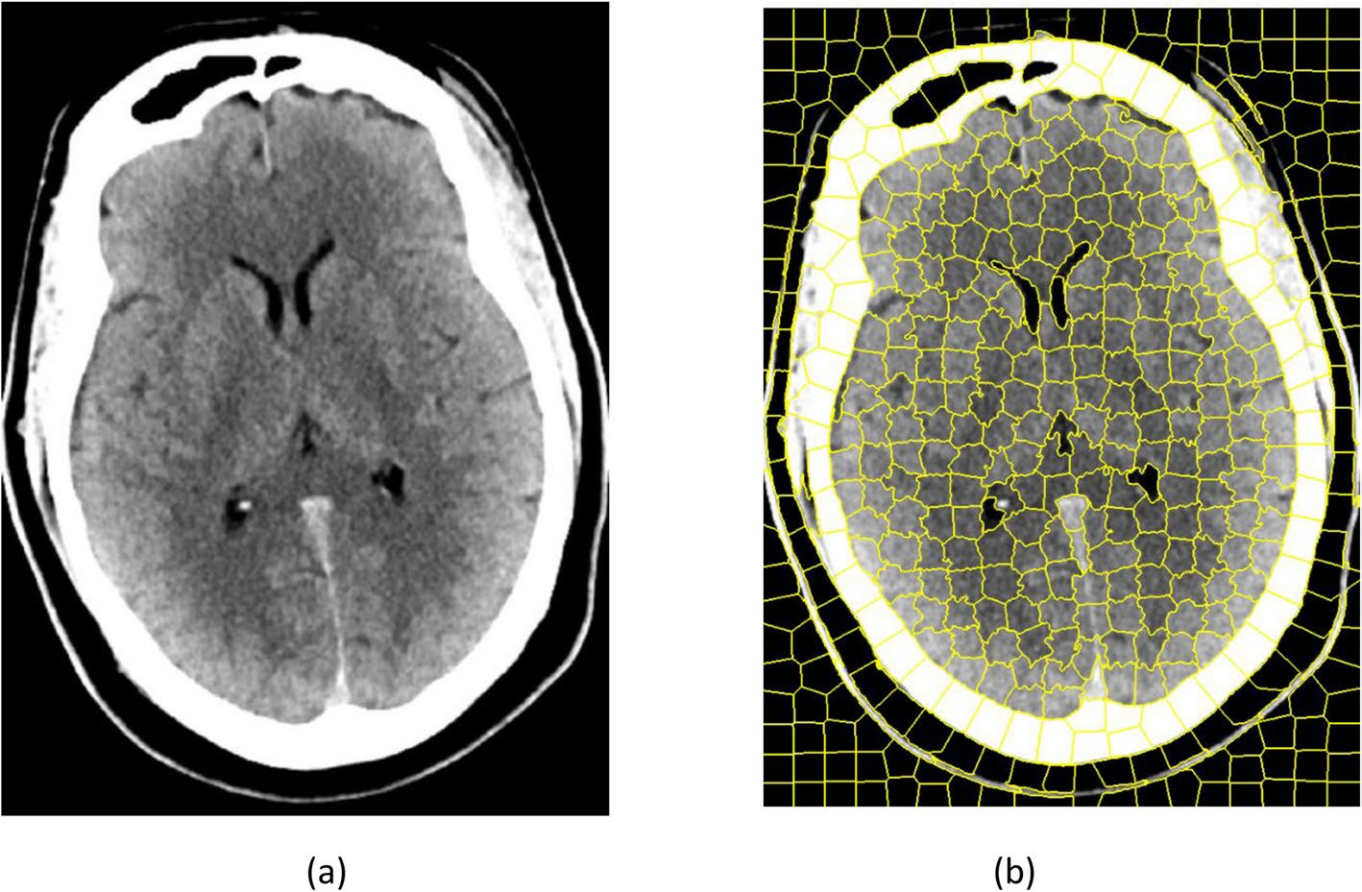
**a** Brain CT; **b** Segmented white matter and gray matter

The drawback of the described segmentation techniques is that the region boundary is not sensitive to the neighborhood. Thus, region-aware algorithms [43, 44] such as linear spectral clustering and simple linear iterative clustering have been proposed. The basic concept behind a superpixel is that adjacent pixels in an image that are continuous and contain similar color intensity and brightness are clustered into a single superpixel, and the region is marked by a boundary. Figure 3 shows superpixel segmentation.

#### Registration

Registration or mapping of a point (*x*, *y*) to another point (*u*, *v*) can be performed using B-splines [45]. Formulated as an approximation problem in a Frennet frame [46] of the target spline, two points under correspondence are made to have the same normal direction. The B-spline surface approximation is formulated as (Eq. 3). *F*_*s*_ is the smoothing term; *p*_*k*_ is the data point, and *x* (*u*_*k*_*,v*_*k*_) are the approximating surface points.

3 $$F=\sum\nolimits_{k}{\Vert x\left({u}_{k},{v}_{k}\right)-{p}_{k}\Vert }^{2}+\lambda {F}_{s}$$

4 $${\Vert x\left({u}_{k},{v}_{k}\right)-{p}_{k}\Vert }^{2}=\sum\nolimits_{k}{\left(\sum\nolimits_{i=1}^{n}{B}_{i}\left({u}_{k},{v}_{k}\right){d}_{i }-{p}_{k}\right)}^{2}$$

**Fig. 4:**
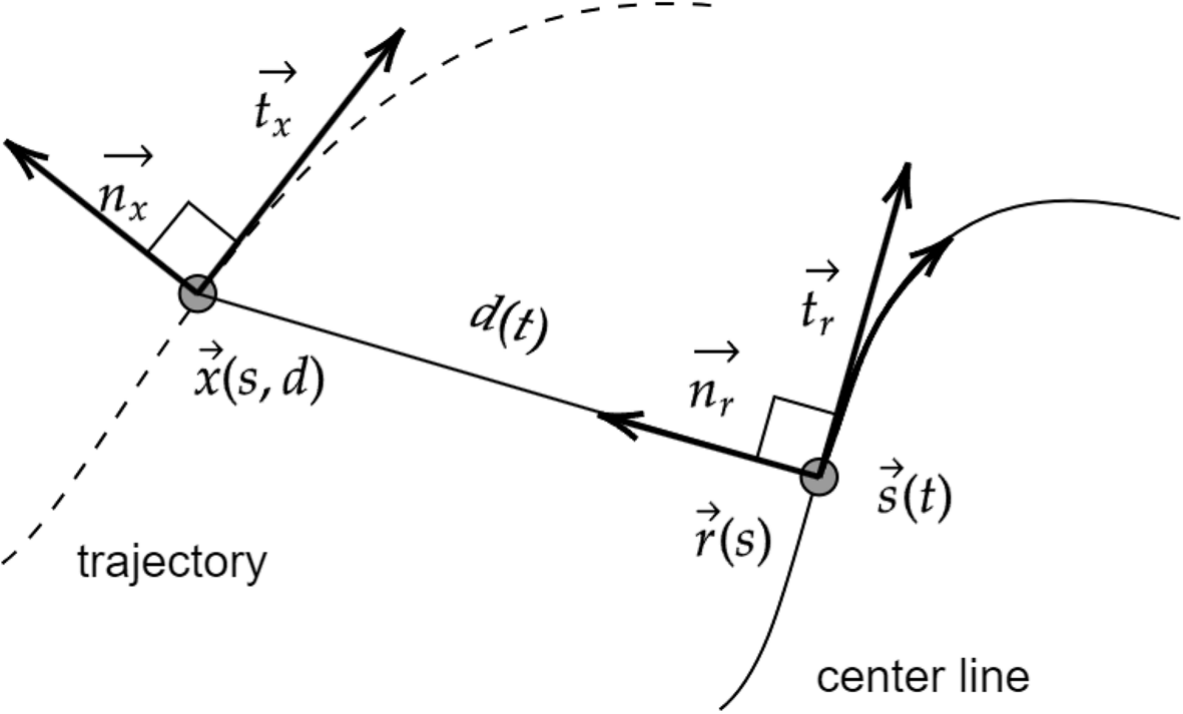
Trajectory curve (dotted) shown as Frennet frame auto-correcting to centerline (solid) for accurate registration

In Eq. 4, ${B}_{i}\left({u}_{k},{v}_{k}\right)$ is a basic B-spline function that is a piecewise smooth polynomial. The B-spline shape can be altered in any direction [47] such that the curve passes through a specific control point *P*. The spline passes through *P* by inserting a knot *u* in *U*, where *U* is the existing set of knots (Eq. 5). Figure 4 shows a set of points in 3D space auto-corrected to a given prior for registration. Figure 4 shows the movement of the curve toward the defined target, and was used for correct registration of images.

5 $$P = \alpha {Q}_{j-1}(u) + (1 - \alpha ){Q}_{j}(u),\alpha \in [\mathrm{0,1}]$$

#### Reconstruction

**Fig. 5:**
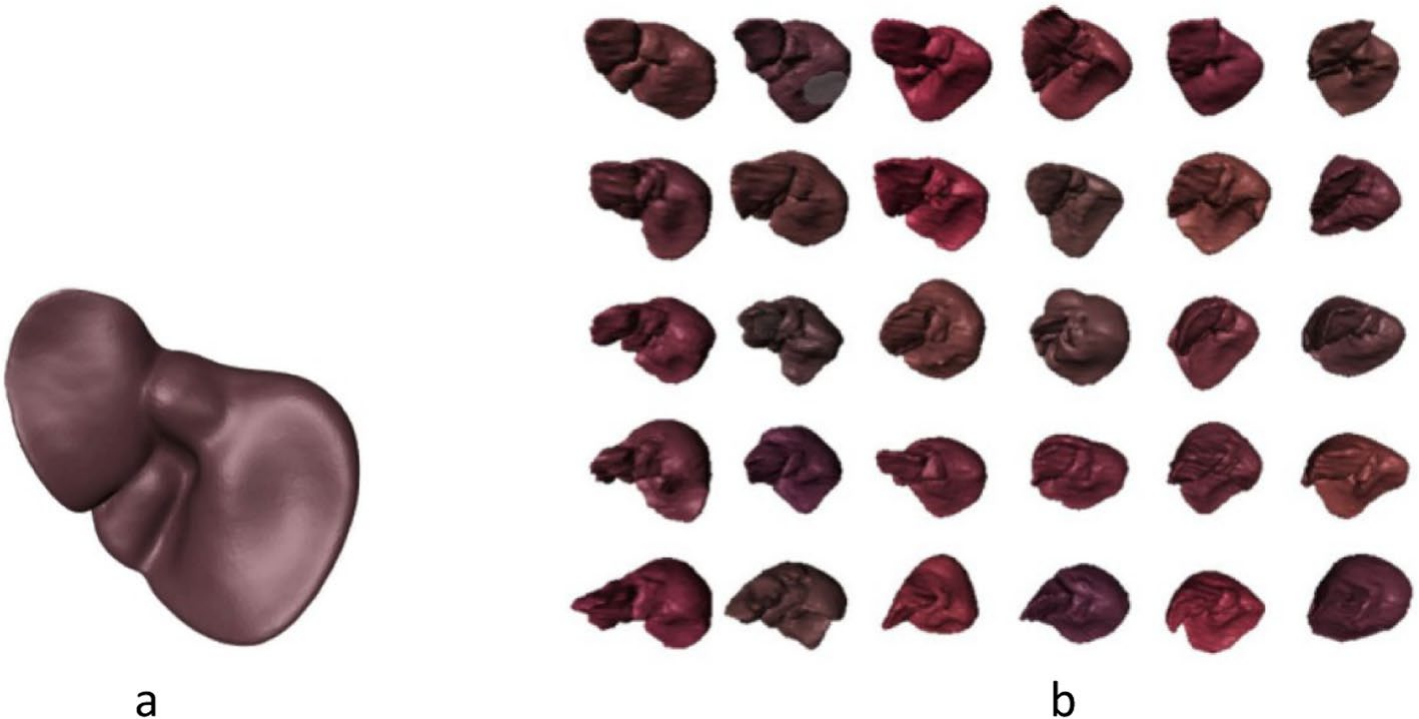
Mean liver model (**a**) and group of reference models (**b**)

The SSM [19] (Fig. 5) is used to establish the correspondence between different 3D shapes of the same organ. The SSM is presented as a linear model in the form shown in Eq. 6.

6 $${V}_{i}=\overline{V }+ {Pb}_{i}= \overline{V }+\sum_{k}{p}^{k}{{b}_{i}}^{k}$$

$\overline{V }$ is the mean shape vector, and *P* = *p*^*k*^ is the matrix of the eigenvectors of the covariance matrix. The rigid transformation is expressed as (Eq. 7):

7 $${T}_{j,min}= \underset{T}{\mathrm{min}}\sum_{k=1}^{m}{\Vert {x}_{1k}- T\acute{x}_{jk}\Vert }^{2}$$

*x*_1*k*_ and *x*_*jk*_ are the coordinates of the two shape vectors. $\acute{x}_{jk}$ is the new vertex location. Then, the final shape *x*_*jk*_ = *T*_*j,min*_* x*´_*jk*_ is the optimum shape with minimum deviation from a reference. In ref. [18], a detailed survey of the use of SSMs was presented; sometimes, the points were closely registered using an elastic registration approach [48] known as the active shape model [49, 50]. Figure 5 shows the mean model of the statistical shape technique.

**Fig. 6:**
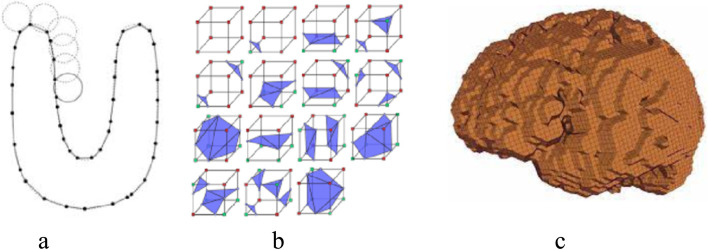
**a** BPA algorithm connecting random points in 3D space; **b** Marching cubes basic building blocks; **c** Output of marching cubes algorithm showing coarse and rough surfaces

The marching cubes [51] algorithm (Fig. 6b) is considered the best for reconstruction of a volumetric model because its algorithm is specifically designed for medical images. In volumetric modeling, details of the texture and layers present under the top surface of the object are required. For surface reconstruction, the ball pivoting algorithm (BPA) [52] (Fig. 6a) is more suitable; only one layer of the surface is required as the output. A limitation in the BPA is that for complete surface reconstruction, the algorithm must be re-iterated with different values of ball radius *ρ*. Another limitation is that the normals of the vertices must be known. However, this is not the case for 2D medical scans. The normal can be estimated using algorithms such as total least squares [53]. Figure 6 shows the conversion of points into connected lines, surfaces, and volumes.

### Reconstruction based on deep learning approach

**Fig. 7:**
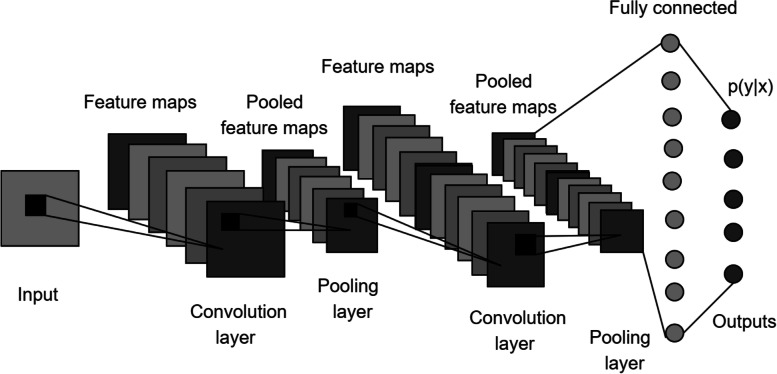
Basic CNN

The traditional approaches (TAs) in Reconstruction based on TA section involve a seed boundary and initialization parameters. This limitation requires a new paradigm for segmentation and reconstruction of medical images. A convolution neural network (CNN), as shown in Fig. 7, is an artificial neural network that accepts a color image with a height of 224 pixels and a width of 224 pixels, beyond which there are convolution layers and other layers including pooling, fully connected, and SoftMax. In a CNN, the number of filters varies in each layer; the filter size can also vary. The filters are modified using a backpropagation algorithm. The weights of the filters are learned and stored for future epochs until the network is properly trained. Pooling layers reduce the feature size by averaging or selecting the maximum within a 2 × 2 neighborhood matrix. Some diagrams indicate the input size and number of filter channels as dimensions at the top of the convolution layers. The fully connected layer flattens the network toward the end and is activated using the SoftMax function. The SoftMax outputs are rendered as classification labels.

Deep learning (DL) methods are state-of-the-art for image-related tasks such as segmentation and registration. The underlying structure of segmentation-based DL models is a CNN. DL methods are modified on top of the CNN such that the features of a set of images are automatically determined by the DL system through experience and training on thousands of samples. Handcrafted features tend to be more time-consuming and inferior in terms of performance compared to DL-supervised methods.

#### Related work

Several versions of CNNs have been proposed to improve model performance. Krizhevsky et al. [54] presented a deep convolution neural network (DCNN) model with eight learned layers: five convolutional layers and three fully connected layers. AlexNet comprises five convolutional layers and three fully connected layers. The implementation is distributed over two graphics processing units. Local response normalization [55] was used as a form of LAT inhibition for competition-based learning. Simonyan and Zisserman [56] presented the visual geometry group networks VGGNet-16 and VGGNet-19. In the VGGNet class of networks, 3 × 3 convolution filters were used with three consecutive fully connected layers with 4096, 4096, and 1000 filters. In VGGNet, there are problems with errors and overfitting. Deeper layers in deep neural networks (DNNs) can incur huge computational costs; in deeper layers, the weights are nearly zero and incur computational loss. Google designed GoogLeNet [57] based on the Hebbian principle. GoogLeNet clusters neurons based on correlation statistics from input images. The previous layers are analyzed for their correlation values and highly correlated neurons are clustered in the next layer. GoogLeNet is more computationally efficient than AlexNet.

**Fig. 8:**
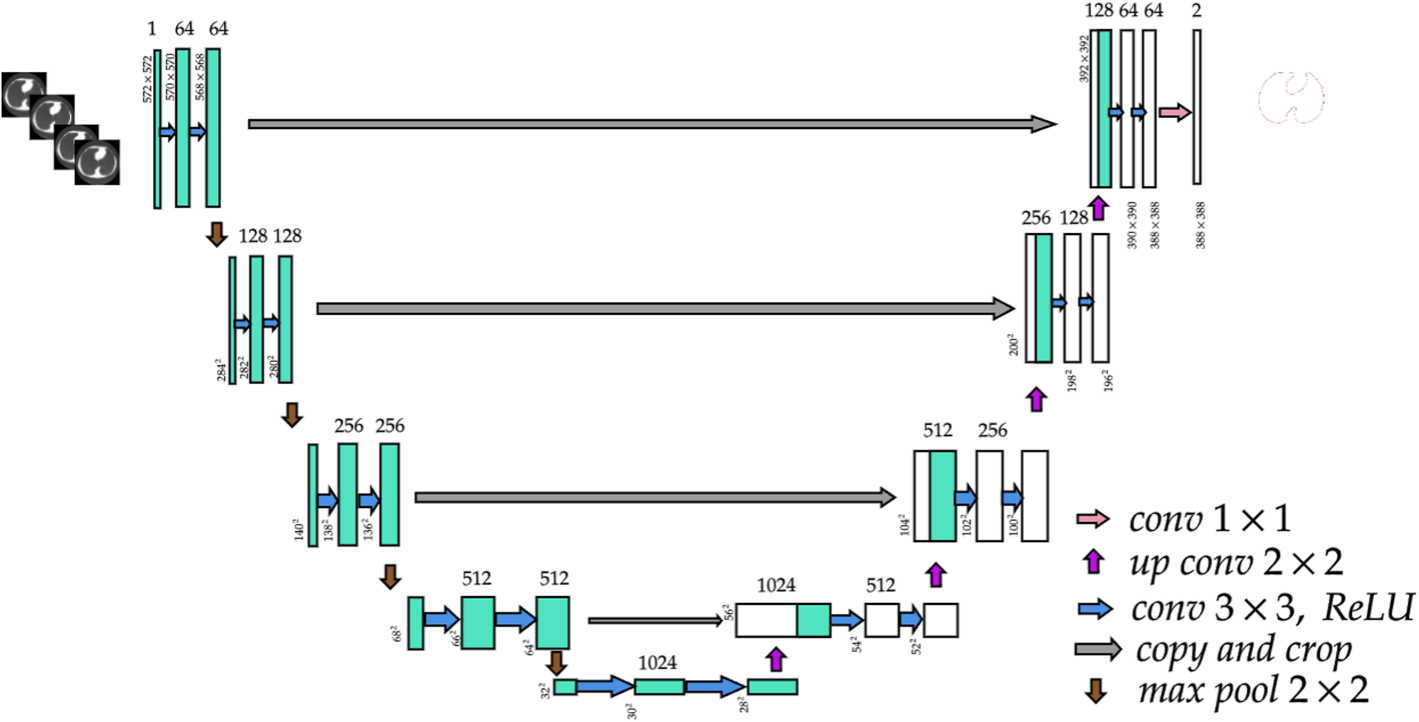
U-Net architecture

To further reduce the computational cost, 3 × 3 and 5 × 5 convolutions were preceded by 1 × 1 convolutions in GoogLeNet V2 and V3. As in a shallow CNN, the number of layers is less than in a DCNN; thus, the network should produce fewer errors as it goes deeper into the network. The network is a copy of a shallow CNN with identity mapping. He et al. [58] proposed ResNet, which skips connections that feed the activation from one layer to another. Google developers further proposed Inception-ResNet [59], in which batch normalization was used only on top of the traditional inception layers to reduce memory consumption; 35 × 35, 17 × 17, and 8 × 8 grids were used for the inception A, inception B, and inception C layers, respectively. Ronneberger et al. [60] proposed U-Net (Fig. 8) as the standard model for biomedical image segmentation. U-Net is trained with fewer examples to achieve high accuracy. U-Net presents symmetric encoding and decoding along with skip connections. Çiçek et al. [61] proposed a modified version of the 3D U-Net for segmentation of 3D volumetric data, in which three planar segments of the ROI in each iteration carefully segmented an anisotropic body.

##### U-Net-based segmentation and other techniques

U-Net [60], as shown in Fig. 8, has been used for medical image segmentation since the early days of DL, originating from the electron microscopy (EM) segmentation challenge. As shown in Table 1, U-Net is ranked higher based on the warping error metric. Istituto Dalle Molle di Studi sull’Intelligenza Artificiale (IDSIA) members used a version of the CNN extending up to 10 layers. The basic idea was to label every pixel and build a classifier that can do this efficiently. The algorithm was submitted to the 2012 IEEE International Symposium on Biomedical Imaging EM challenge, and was the best-performing algorithm.

**Table 1:** Performance of U-Net in the EM challenge (March 6th, 2015) [60]

Rank	Group name	Warping error	Rand error	Pixel error
1	U-Net	**0.000353**	0.0382	0.0611
2	DIVE-SCI	0.000355	0.0305	0.0584
3	IDSIA [62]	0.000420	0.0504	0.0613
4	DIVE	0.000430	0.0545	**0.0582**
$$\vdots$$				
10	IDSIA-SCI	0.000653	**0.0189**	0.1027

In Medical image segmentation before U-Net and Machine learning (ML)-based medical image segmentation sections, earlier automated medical image segmentation techniques are discussed.

##### Medical image segmentation before U-Net

Sharma and Aggarwal [63] classified segmentation techniques based on amplitude, edge, and region. These methods are categorized as gray-level-dependent techniques. Edge-based techniques include edge relaxation [64], the border detection method [65], and the Hough transform [66, 67]. Other unsupervised methods are based on k-means [68], hard C-means, and fuzzy C-means (FCM). A review of FCM segmentation was presented in ref. [69]. In ref. [70], medical image segmentation was performed by detecting image features using a feature extraction method based on stacked independent subspace analysis [71] combined with PCA to match small patches using feature matching steps such as the histogram of gradient. In ref. [72], use of RG algorithms was proposed as a segmentation technique and for feature extraction, including Zernike moments [73] with a CNN.

##### ML-based medical image segmentation

Cascaded networks [74, 75] are expensive for evaluating regions within and outside the target object. One layer of the cascaded network roughly estimates the ROI after rejection of redundant information. As the name suggests, region evaluations are based on the inputs received from preceding cascaded networks. The current layer reevaluates the ROI and rejects the outside boundary before forwarding it to the next network. To improve on cascaded networks, Li et al. [76] presented a lightweight regression network. Their method was improved; their main architecture was inspired by U-Net. Torbati et al. [77] used a moving-average self-organizing map. Weight vectors moved toward the filtered outputs after the trace matrix was updated, depending on the winning neuron and neighboring functions. Ultimately, clustered neurons corresponding to the topographical nodes were segmented. Al-Ayyoub et al. [78] demonstrated accelerated lung CT segmentation using brFCM [79] over the earlier FCM. A Markov random field (MRF) [80] was used for brain-image segmentation. The MRF is a powerful tool for representing an image as a stochastic model based on color intensity values. Deng and Clausi [81] proposed a new unsupervised image segmentation model built from a simple MRF; in the expectation-maximization algorithm, the parameter *α* was allowed to update, eliminating region-labeling and feature-labeling modeling confusion. Monaco and Madabhushi [82] introduced class-specific weights to the maximum a posteriori and maximum posterior marginal estimation criteria.

##### Neural network frameworks

**Fig. 9:**
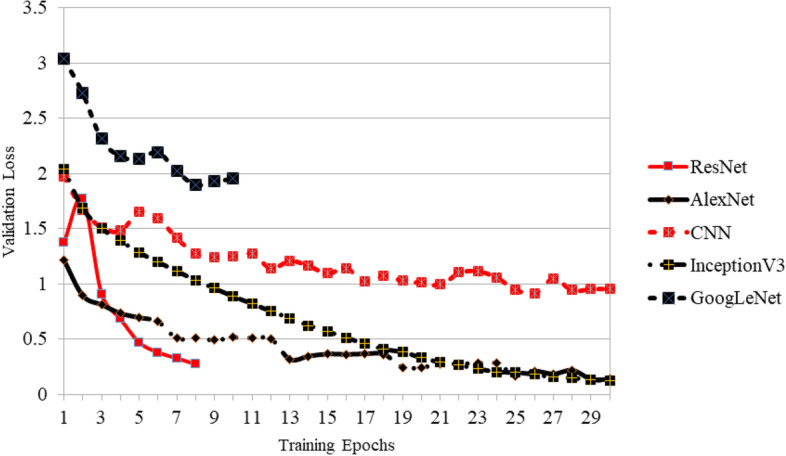
Validation loss in classifying Cifar-10 dataset

In this study, basic bare DNN models were tested on simple image classification tasks using the Kaggle Cifar-10 dataset. The results are shown in Fig. 9. Application of these DL methods to 3D reconstruction of 2D medical images is presented in Table 2.

**Table 2:** DL methods for 3D reconstruction

Reference	Contribution
DeepOrganNet [83]	Multiple meshes from a single view medical image
AlexNet [84]	Use of PReLU activation function
Jeyaraj and Nadar [85]	DCNN to improve medical image reconstruction and identify complex morphological regions
Shakarami et al. [86]	Improved AlexNet-SVM for feature extraction and classification
Xie et al. [87]	GoogLeNet based post processing steps to remove artifacts in sparse-view CT reconstruction
Islam et al. [88]	Using DCNN to classify 3D organ that is rotation and translation invariant
Ke et al. [89]	VGGNet trained model on Epileptic EEG classifier
Seol et al. [90]	ResNet50 based network for bone fracture diagnosis
Li and Shen [91]	3D Unet based Neuron reconstruction

Modern neural network-based learning is based on application of a CNN. Mask R-CNN [92] is a framework for object instance segmentation. This method extends the faster R-CNN. The boundary-preserving Mask R-CNN [93] contains a boundary-preserving mask head, in which the object boundary and mask are mutually learned via feature fusion blocks. A mesh R-CNN [94] is built on top of Mask R-CNN with a mesh prediction branch that outputs meshes with varying topological structures through coarse voxelization; they are converted to meshes and refined using a graph convolution neural network (GCNN) [95]. Vox2Mesh [96] converts an occupancy grid into a mesh. Because a voxel grid lacks fine geometric details, the selected grid is converted into a triangular mesh. Pixel2Mesh [97] is a DL architecture that produces a 3D shape in a triangular mesh from a single-color image, and is implemented on top of a GCNN.

##### Point-cloud-based reconstruction applications

Some methods [98, 99] use point clouds for fully automatic surface reconstruction [23]. The problem of reconstruction is formulated as a point-cloud completion problem, as in a point completion network [100]. The objective is to reconstruct a noisy or partially missing point distribution.

8 $${L}_{total} = {L}_{coarse} + \alpha *{L}_{dense}$$

**Fig. 10:**
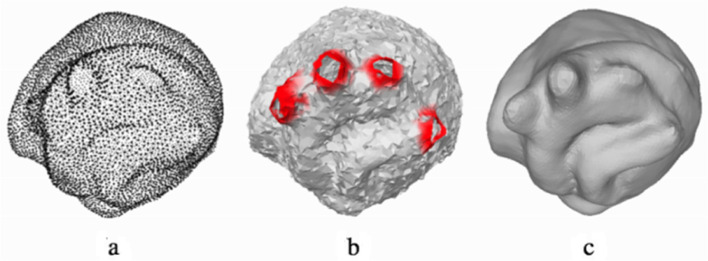
**a** Point cloud; **b** Rough surface; **c** Final mesh

In Eq. 8, *L*_*coarse*_ is the point cloud from the points on the contour boundary. *L*_*dense*_ enforces smoothness at the local and global levels. *α* is the weight factor and acts as the controlling term [101]. Figure 10 shows the completion of the points in the final mesh.

## Overview of recent developments in organ-specific 3D reconstruction in medical diagnosis

### Lungs

Sun et al. [49] used CUBIC and 3D imaging of solvent-cleared organs over CT scans or MRI to avoid inhaled hydrogen atoms administered to image 3D mouse lungs and used Nissl-staining [102] for rendering 3D lung structures. Bruno and Anathy [103] found that more attention was required for 3D reconstruction of ER-mitochondrial interactions during the initial phase of lung disease. Durhan et al. [104] stressed the importance of contrast-enhanced CT for 3D reconstruction. Filho et al. [105] proposed an adaptive crisp ACM 2D to diagnose pulmonary disease by lung segmentation, and used the Open GL API for visualization. Li et al. [106] proposed the use of a geometric active contour model to segment lungs in a single algorithm using a supervised segmentation method. This approach yielded faster segmentation than the coarse-to-fine method. Le Moal et al. [107] reported that medical diagnosis was performed after uploading patient CT scans to the Visual Patient*™* server and downloading the 3D model. This approach is costlier; its impact on surgical efficiency is currently under investigation. González Izard et al. [108] proposed development of the NextMed platform to convert DICOM images into 3D models and visualizations, including segmentation, a tedious manual task. Joemai et al. [109] reported that CT reconstruction devices over filtered back propagation produced less similarity with ground truth images than the forward-projected model-based Iterative Reconstruction Solution and Adaptive Iteration Dose Reduction in three dimensions. Pereira et al. [110] addressed lung issues using MRI imaging technology based on 3D-ultra-short echo time to obtain morphological and functional images of the lungs over traditional 2D image registration techniques. Wang et al. [97] proposed an automatic 3D classification framework built on top of an R-CNN to predict pre-invasive or invasive lung cancer to facilitate selection of proper treatment. Jin et al. [111] proposed a generative adversarial network (GAN)-based synthetic lung nodule generation for improved CT datasets by introducing a novel multitask reconstruction loss term to generate more realistic and natural nodules. Furumoto et al. [112] proposed using fluorodeoxyglucose-PET/CT combined with high-resolution CT on the solid part of a tumor for better prediction than using only CT to identify clinical stage IA adenocarcinoma. Grothausmann et al. [113] generated a 3D reconstruction of the alveolar capillary network in the lungs using 2D histological serial slices. Morales-Navarrete et al. [114] proposed use of fluorescent markers in high-resolution microscopic images to digitally reconstruct 3D representations of cells in tissues and their critical subcellular parts.

### Knee

Kasten et al. [115] proposed 3D reconstruction of knee bones using a CNN from two biplanar x-ray images. Ciliberti et al. [116] proposed 3D reconstruction of knee joints to assess the relationship between bone mineral density and cartilage status. Hess et al. [117] performed 3D reconstruction of knee joints to assess the alignment of the femur and tibia in osteoarthritis ailments using KNEE-PLAN® software. Wu and Mahfouz [118] proposed 3D representation of the knee using a single fluoroscopic x-ray based on a nonlinear SSM to design a patient-specific instrument for total joint replacement. Bao et al. [119] used MIMICS 10.01 software on a 64-slice spiral CT scan to determine the postoperative range of motion. Marzorati et al. [120] used DCNN-based segmentation of the femur and tibia, and evaluated the impact of segmentation uncertainty on surgical planning for personalized surgical instruments.

### Kidney

Puelles et al. [121] used confocal microscopy for 3D imaging of kidneys up to a depth of 50-80 µm, with new advances in clearing methods to remove lower-order lipids and pigments in tissue to render it as transparent. Guliev et al. [122] proposed simple 3D reconstruction of the kidney pelvicalyceal system (PCS) through semi-autonomous PCS segmentation. Mercader et al. [123] used a 3D-printed model and CT scan images for better surgical planning for a horseshoe kidney. Chaussy et al. [124] used 3D-slicer software to semi-automatically segment 14 scans from 12 patients with Wilms’ tumors. The mean segmentation was 8.6 h. Les et al. [125] proposed use of parametric coefficients to determine the location of the kidney in a CT image.

### Liver

Chen et al. [84] proposed a serial encoder-decoder (SED) DL approach. Two SEDs were used: the first to segment the ROI, and the second to further segment the results obtained from the first network. The SED was designed using a U-Net architecture. Yeo et al. [126] proposed use of 3D reconstruction of 2D CT and MRI images as a tool for training medical students in liver tumor resection. Fang et al. [127] validated 3D reconstruction of the liver in the pre-operative stage; intra-operative and post-operative stage scores were assigned based on whether 3D visualizations were helpful; the 3D visualizations produced a high score for each phase. Tatamov et al. [128] reported that use of 3D visualization in liver laparoscopic herpetology reduced the risk of intra-operative complications due to bile and paralytic cysts. In a study by Fang et al. [129], eye observations were used for intra-operative lesion identification. 3D visualization, as an intelligent imaging tool and diagnostic medium, helped solve problems using traditional methods in intra-operative stages.

### Brain

Bjerke et al. [130] proposed developing a reference atlas for the rodent brain as part of the Human Brain Project and built reference atlases for the human brain as part of the BigBrain Project. Ebner et al. [131] compared the localization and segmentation of the fetal brain to manual segmentation considering the constant motion of the fetus in the prenatal stage between fast 2D scan slices. Du et al. [132] proposed a dilated encoder-decoder network to improve the MRI 2D image resolution using 3D dilated convolution.

### Miscellaneous

For medical diagnosis, exposure to harmful CT rays during intra-operative stages is not desirable, and it is not possible to monitor organ status through CT scans in patients undergoing operative procedures. Thus, x-raying the area of interest and projecting instantaneous 3D reconstructions is most desirable. Use of a single x-ray image is more desirable; however, training a CNN with a single image requires a different approach. As described in ref. [12], a GAN [133] was used to reconstruct 3D bone structures using DRR. Using oral x-rays for 3D reconstruction, panoramic x-ray [134] imaging providing a linear view of the oral cavity to observe artifacts can be used to restore the mandible; 3D alignment was performed with the dental arc using the contour extraction method on the original dental structure. Such deformable shapes from two dimensional x-ray imaging are a key achievement, as described in ref. [135]; an image-to-graph convolution network (IGCN) using deformation mapping by point-to-point correspondence was proposed. The IGCN was designed as an organ-independent network.

From angiogram CT scans of older patients, 2D DRRs were generated to simulate real-world x-rays, instead of patients switching between the two devices. In ref. [115], a biplanar x-ray imaging approach was described for 3D reconstruction using supervised cross-entropy weight loss. The first loss was measured as the distance between each voxel and the surface tissue (ground truth); the second loss was the unsupervised reconstruction loss. In ref. [136], a neural network was trained using a triplet loss function to identify normal and deformed bones to relate the most closely predicted bone shape to a predefined set of bone shapes.

### Performance comparison of state-of-the-art models

A detailed survey was reported in ref. [137], from which some networks were selected; their performances are compared in Table 3. The chosen networks and models indicate the most relevant recent work. The modality of the image input was not a selection criterion; irrespective of whether the data source was single- or multi-slice, some of the models were retained. Some of the models were based on supervised learning [138–144]; others were unsupervised [145–147].

**Table 3:** State-of-the-art methods, performance and objective

Model	Metric	Result	Objective
DIRNet [138]	DICE	0.80 ± 0.08	Cine MRI registration
CNN + STN [139]	SNR	207.42 ± 96.73	Tolerating respiratory motion while imaging
Unet + STN [140]	DICE	0.90 ± 0.60	Deformable medical image registration
RegNet [141]	MAE	1.19 ± 1.17	Non rigid image registration
LungRegNet [142]	TRE	1.59 ± 1.58	Unsupervised deformable image registration
SARNet [143]	DICE	0.741 ± 0.08	Data independent image registration
3D Unet + SVF [144]	DICE	0.6794 ± 0.04	Spatio-temporal regularizer
VoxelMorph [145]	DICE	0.78 ± 0.03	Deformable medical image registration
GAN + AIRnet [146]	D-score^a^	0.60	Adversarial image registration
PDDNet [147]	DICE	0.584 ± 0.059	Probalistic dense displacement network

^a^Discrimination score

9 $$DSC = \frac{2TP}{2TP+FP+FN}$$

Performance was measured using DICE [148] (Eq. 9) for the segmentation quality, where TP is the percentage of pixels labeled as true positives (positive in test results and positive in ground truth); TN is the percentage of pixels labeled as true negatives (marked as outliers or noise in both test results and ground truth); FP is the percentage of pixels labeled as false positives (marked as positive in test results and negative in ground truth); FN is the percentage of pixels labeled as false negatives (marked as negative in test results and positive in ground truth). Other metrics used included the signal-to-noise ratio, mean absolute error, discrimination score, and target representation error.

### Pros and cons of DL and TA

3D reconstruction from 2D medical images is subject to time and data availability. Traditional methods have been used to obtain faster satisfactory results. However, as modern-day computation shifts toward a new paradigm of automation, the success of DL methods has been observed in other areas of 3D reconstruction. Thus, the DL approach has seeped into medical organ reconstruction and is the way of the future. Several subtasks, from segmentation to registration and smoothing, can also be performed using modern DL methods. However, these methods are sensitive to the input labels and are typically supervised. The final outcome of a DL approach is superior to that of TAs but is constrained by the size of the training examples. TAs require human intervention and are not automatic, as are some DL methods. Table 4 presents several strategies and their advantages and disadvantages in performing common 3D reconstruction tasks.

**Table 4:** Pros and cons of DL and TA in 3D reconstruction of medical images

Model	Type	Objective	Pros	Cons
Active Contour [39]	TA	Segmentation and contour detection	Reset and re-evaluation is fast, dynamic and verifiable with a GUI	Requires initial seed region, for correct segmentation more than one trials of seeds at proper location is needed, human supervision is needed
U-Net [61]	DL	Segmentation and contour detection	Does not require initial seed region, highly accurate and much superior to TA, automatic feature detection, available as pre-trained model	Accuracy is dependent on training examples, incurs huge computation cost, addition of new data is difficult and requires fine tuning, initial target masks are required
Marching Cube [52]	TA	3Dmesh generation	Easy to implement, tailor made for medical image reconstruction, requires very little storage of memory	Generated output is rough and not smooth, higher volume of CT slices generates higher level of details, requires complete set of segmented volume
DNF-Net [149]	DL	3Dmesh generation	Filters noisy polygon normal, accurately predict orientation and direction of surface normal, superior to TA	Insufficient training examples results in unexpected results, does not generate mesh by itself, requires update algorithm to convert normal into mesh
B-Spline surfaces [45]	TA	2D-3D registration	Easy to implement, requires no prior training examples	Requires equal number of control points from every CT scan within a volume, human intervention is needed
Voxelmorph [145]	DL	2D-3D registration	Automatic and unsupervised	As an atlas based approach it is reference dependent

## Summary on current trends

Most reconstruction studies have focused on deformable registration and segmentation. The DL approach has outperformed traditional semi-autonomous methods. The most common is U-Net for segmentation and CNN, and its variants for registration. DRR [15] is also gaining popularity due to the lack of large-scale medical image databases for 3D reconstruction. For registration, large deformation diffeomorphic metric mapping [150] is a metric; for the final surface reconstruction, the Dice score and structural similarity index measure score are commonly used to measure the outcome. In addition, the mean square error and average surface distance are used to assess prediction accuracy. Expected future trends and scope are presented in Table 5.

**Table 5:** Recent trends and research gaps for further research

Objective	Techniques	Reference	Year	Limitations/research gap
3D mesh compression	DCT based source image compression	[151]	2022	Direct compression on a 3D model has not been performed
	Training neural networks with signed distance function and network weights as a field	[152]	2022	The method is not lossless as mapping weights back to coordinates is not reversible
3D mesh generation	Recurrent neural network (RNN)	[153]	2022	Supervised learning and requires training labels for target normal. There is still a gap in unsupervised polygon normal regression
	Deep normal filtering neural network	[149]	2020	
3D mesh encryption	Encryption using chaotic behavior in edge computing devices	[154]	2023	Even though vertices are encrypted the edges remain connected and limits model security
	Using correlation between two sets of vertices to recover encrypted data	[155]	2022	
3D mesh smoothing	RNN	[153]	2022	These methods either correct the vertices or correct the face normal. These methods do not output a final mesh with vertices and labelled edges
	Deep normal filtering neural network	[149]	2020	
Medical scan segmentation	U-Net	[63]	2015	Supervised learning and requires training labels for target masks. There is a huge gap in unsupervised DL based segmentation
	Attention U-Net	[156]	2022	
Real time 3D imaging based prediction and diagnosis	Spatio-temporal long short term memory	[157]	2022	Real time 3D model of a moving heart is not constructed in this paper. Faster and efficient way of 3D organ modeling needs to be studied as normal heart rate is 60–100 bpm. So each model should not be modeled fast

Current trends include effective 3D mesh-compression techniques, 3D mesh encryption, 3D mesh generation, medical image segmentation, 3D mesh smoothing, and real-time 3D-image processing. Compression techniques involve compression of source images to generate a concise model. However, direct compression of a 3D mesh can also be studied. 3D mesh encryption is important owing to the active role of IT-enabled services in the healthcare sector. Medical-image segmentation is an old problem; however, unsupervised segmentation using an untrained DNN has not yet been achieved. This application reduces the training time, increasing real-time error-free reconstruction for better training and analysis. Vital organs such as the heart, lungs, and arteries change shape continuously. Fast reconstruction is the only solution for real-time visualization and virtual reality-enabled services.

## Major challenges

The major challenge in 3D reconstruction is image preprocessing [158]. Conventional image cleaning can omit many data points from an image; however, it can help accurately depict tissues and organs. Removing unwanted pixels such as noise is also important. Noise can cause problems in selection of the proper segmentation area, especially in cases where the left and right lobes appear to constantly touch each other in some slices in small regions after dilation and erosion operations, whereas in the ground truth, they may not. Handling unstructured point-cloud data without the related topological information is another challenging problem. These data points are scattered densely or sparsely, requiring specialized algorithms for processing. Another major limitation is the availability of 3D medical models such as ShapeNet [159] and ModelNet [160] for validation. One reason for the lack of such public datasets is that hospitals and patients are unable to obtain permission to make health records publicly available. Most datasets available for 2D medical images are concerned with larger organs; more work is required to create benchmark and gold-standard datasets.

## Conclusion and future work

3D reconstruction is becoming more important in medical assistance; its application in medical science is promising. Many areas of concern remain as there is no well-defined or gold-standard dataset for 3D human organs. Thus, GAN-based DL models were used to recreate the synthetic datasets. Although SSM-based models perform well and have been studied, their application is becoming less frequent.

3D reconstruction is expensive; with the requirement of higher-resolution images, use is confined to specially designed medical devices. The motivation for research in this area is to make 3D reconstruction affordable and less time-consuming without compromising accuracy. The most common enhanced radiological methods for day-to-day diagnosis are CT, MRI, and their variations.

In the future, 3D reconstruction is expected to become a more common method of reporting to patients. Further research will include new methods of model security, compression, representation formats, holographic and virtual reality-based representations, real-time representations in operative procedures such as endoscopy, and 3D representations at the cellular level that allow lower-calibrated microscopes to efficiently observe cell structures in largely magnified 3D model representations.

## Data Availability

Not applicable.

## Abbreviations

3D: Three-dimensional
2D: Two-dimensional
ACM: Active contour model
PCA: Principal component analysis
PCS: Pelvicalyceal system
PET: Positron emission tomography
BPA: Ball pivoting algorithm
RNN: Recurrent neural network
CNN: Convolution neural network
ROI: Region of interest
CT: Computed tomography
DCNN: Deep convolution neural network
SED: Serial encoder-decoder
DL: Deep learning
DNN: Deep neural network
DRR: Digitally reconstructed radiograph
EM: Electron microscopy
FCM: Fuzzy C-means
SSM: Statistical shape model
GAN: Generative adversarial network
HU: Hounsfield unit
IDSIA: Istituto Dalle Molle di Studi sull’Intelligenza Artificiale
LAT: Lateral
MRF: Markov random field
MRI: Magnetic resonance imaging
GCNN: Graph convolution neural network
IGCN: Image-to-graph convolution network
RG: Region growing
TA: Traditional approach
